# Supplementary material for: Sustained Activation of the Anterior Thalamic Neurons with Low Doses of Kainic Acid Boosts Hippocampal Neurogenesis
Source: Cells. 2022 Oct 28;11(21):3413. doi: 10.3390/cells11213413 (PMC9655699; doi:10.3390/cells11213413)
Supplement: Supplementary file 1 [file cells-11-03413-s001.zip › cells-1973901-supplementary.pdf]

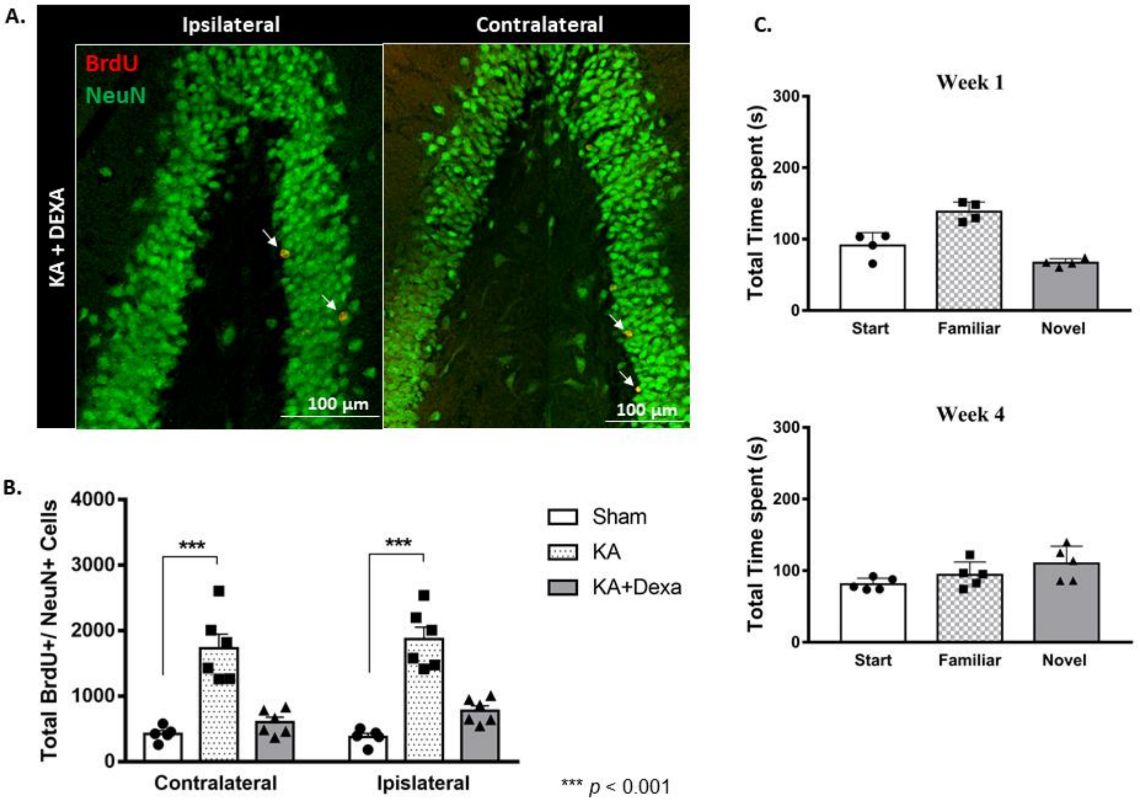

Figure S1. Daily treatment with dexamethasone prior to/and simultaneous with KA maintained neurogenesis and exploration at near-sham levels. (**A**) Confocal images showing immunofluorescence labeling of NeuN and BrdU (arrows) in the DG of KA + Dexa group at 4 weeks. Images were taken as Z stacks using 40X-oil objective. (**B**) Stereological quantification of BrdU-labeled cells in the DG of sham (n = 5), KA (n = 6) and KA + Dexa (n = 6) injected groups at 4 weeks. Determination of statistical significance of differences was made using two-way ANOVA followed by Sidak's multiple comparison test. (**C**) Graph representing the mean ± SEM of the total time spent in the novel, familiar (second arm) and start arms at week 1 and after 4 weeks following KA + Dexa. Repeated measures one-way ANOVA followed by Tukey's multiple comparison was performed to determine statistically significant differences in the total time spent between the different arms.
